# Demonstration of a family of X-ray dark-field retrieval approaches on a common set of samples

**DOI:** 10.1107/S1600577525011403

**Published:** 2026-01-22

**Authors:** Samantha J. Alloo, Ying Ying How, Jannis N. Ahlers, David M. Paganin, Michelle K. Croughan, Kaye S. Morgan

**Affiliations:** a School of Physics and Astronomy Monash University Australia; b Research Group Biomedical Imaging Physics, Department of Physics, TUM School of Natural Sciences & Munich Institute of Biomedical Engineering Technical University of Munich Germany; University of Malaga, Spain

**Keywords:** X-ray dark-field, speckle-based imaging, propagation-based imaging, single-grid imaging

## Abstract

There are various imaging setups capable of capturing dark-field images, each with its own capabilities and limitations. Across all these setups, the underlying physical mechanism responsible for dark-field contrast is generally defined similarly: small-angle X-ray scattering from sub-resolution structures, multiple refraction from large resolvable features and/or edge effects. Despite this conceptual consistency, there remains a lack of studies that directly compare different dark-field imaging setups using identical samples. We have recently developed a ‘family’ of dark-field imaging techniques that, although they have different experimental setups, each extract dark-field contrast by directly resolving dark-field-associated local image blurring with a high-resolution camera. In this paper, we provide a qualitative comparison of the capabilities of this family of methods, covering both different experimental acquisition methods and image-retrieval algorithms for this subset of dark-field imaging techniques. We acquired X-ray imaging data from two test samples using synchrotron propagation-based, single-grid and speckle-based setups, and retrieved dark-field images from each dataset using an appropriate algorithm. The dark-field retrieval methods differed in their computational framework: single-shot algorithms were applied for the single-grid and speckle-based approaches, an additional multi-exposure method was used for speckle data, and a dual-energy method was applied to the propagation-based data. We find that all approaches successfully retrieve dark-field contrast in microstructure-dense regions, as expected. Differences arise at high-spatial-frequency sample features, such as edges, and some approaches exhibit artefacts. We attribute these differences to variations in experimental parameters (*e.g.* pixel size, sample-to-detector distance and X-ray energy) and to the algorithmic assumptions underlying each technique (*e.g.* the single-material assumption and treatment of phase effects). This study aims to guide users of dark-field imaging in selecting the most suitable technique for their imaging goals. To this end, we provide a summary table and highlight opportunities for future research into the sources of dark-field contrast across emerging methods.

## Abstract 2

This study aims to guide users of dark-field imaging in selecting the most suitable technique for their imaging goals. To this end, we provide a summary table and highlight opportunities for future research into the sources of dark-field contrast across emerging methods.

## 1. Introduction

X-rays interact with any sample they pass through, and measuring these sample-induced modifications to the X-ray wavefield enables an image to be formed. Conventional attenuation-based X-ray imaging measures the X-ray transmission through an object to form an image and is most suitable for objects with strongly varying densities. X-rays also change direction, or shift in phase, when passing through an object, and exploiting this effect provides phase-contrast X-ray images. First realized six decades ago (Bonse & Hart, 1965 ▸), phase-contrast X-ray imaging can visualize low-density structures that are invisible in conventional attenuation images (Lewis, 2004 ▸) and/or offer radiation dose savings without compromising image quality (Gureyev *et al.*, 2017 ▸).

The most recently developed X-ray imaging modality is dark-field imaging, where contrast is generated from X-ray scattering caused by unresolved sub-resolution structures within the sample (Pagot *et al.*, 2003 ▸; Pfeiffer *et al.*, 2008 ▸). Dark-field contrast is typically seen as a result of small-angle X-ray scattering or multiple refractions of the incident X-ray beam (Pfeiffer *et al.*, 2008 ▸; Yashiro *et al.*, 2010 ▸; Magnin *et al.*, 2023 ▸), but sharp features like edges can also generate dark-field contrast (Yashiro & Momose, 2015 ▸; Alloo *et al.*, 2025 ▸). Dark-field-generating structures cannot typically be resolved using attenuation-based or phase-contrast imaging, but the scattering from these structures can be detected using specialist X-ray imaging setups, often the same ones used for phase-contrast imaging. Dark-field imaging provides unique capabilities unavailable in other X-ray modalities, for example providing access to information about lung alveoli, the air sacs that make up the lung, which are not resolved in conventional attenuation-based chest imaging (Zimmermann *et al.*, 2022 ▸; Urban *et al.*, 2023 ▸).

Capturing dark-field X-ray images involves two steps: (i) acquiring intensity data using a dark-field-sensitive setup and (ii) applying an image-retrieval algorithm to separate and extract the dark-field signal from other contributions to image contrast, such as attenuation and phase, that are present in the intensity data. The second step, image retrieval, typically yields not only a dark-field image but also complementary attenuation and phase images, although this paper focuses solely on the dark-field retrieval aspect. However, it is worth noting that, in high-resolution imaging, retrieving the dark-field signal can improve the resolution and accuracy of phase/attenuation reconstructions compared with when dark-field effects are omitted (Leatham *et al.*, 2023 ▸; Ahlers *et al.*, 2024 ▸; Alloo & Morgan, 2025 ▸).

**Figure 1:**
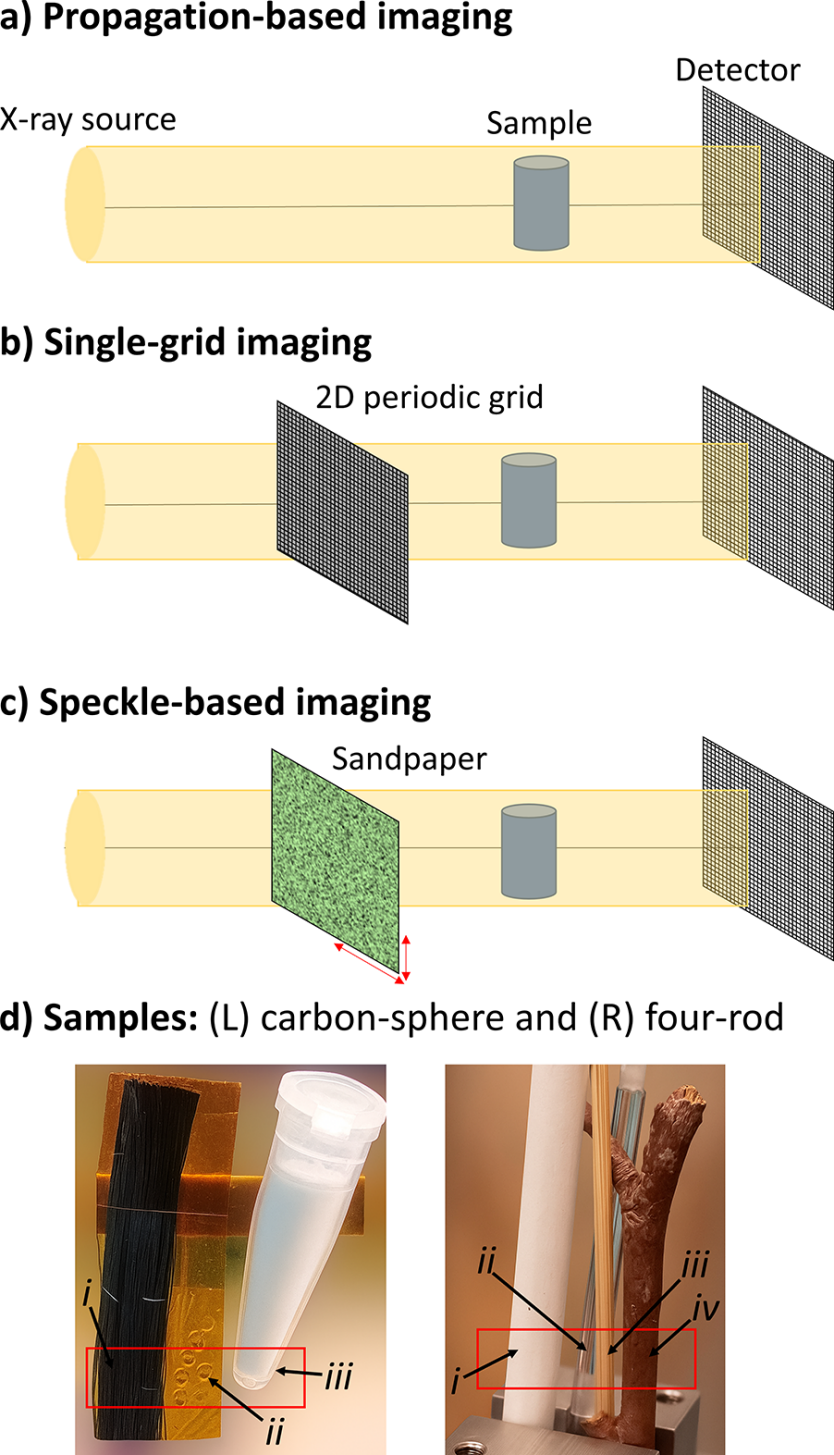
Experimental setups for the subset of dark-field X-ray imaging techniques explored in this study. (*a*) Propagation-based imaging, (*b*) single-grid imaging, which employs one 2D periodic grid, and (*c*) speckle-based imaging, which uses a piece of sandpaper or random membrane. (*d*) The two samples imaged using the setups shown in panels (*a*)–(*c*), with the red boxes indicating the regions imaged. The carbon-sphere sample comprised (i) carbon fibres, (ii) 1.5 mm diameter polymethyl meth­acrylate (PMMA) spheres and (iii) 6 µm diameter PMMA microspheres in a plastic tube. The four-rod sample consisted of (i) a reed diffuser stick, (ii) a solid PMMA rod, (iii) a toothpick and (iv) a tree twig.

There are several well established X-ray imaging setups capable of capturing dark-field images, including grating interferometry (Pfeiffer *et al.*, 2008 ▸) and edge illumination (Olivo & Speller, 2007 ▸; Endrizzi *et al.*, 2014 ▸). Grating interferometry can achieve large-area imaging, as its setup is compatible with large-area large-pixel detectors, with applications in clinical radiography (Tanaka *et al.*, 2013 ▸; Momose, 2020 ▸; Willer *et al.*, 2021 ▸) and computed tomography (Viermetz *et al.*, 2022 ▸), with dark-field contrast particularly helpful in lung imaging. Edge illumination is also suitable for large samples and has made progress towards security applications, with dark-field contrast providing the ability to differentiate between materials for explosives detection (Partridge *et al.*, 2024 ▸). Edge illumination has also been applied to detecting micrometre-sized cracks in thin solar cells for quality assurance (Wieghold *et al.*, 2019 ▸). Among the newer dark-field-sensitive imaging setups are propagation-based (Gureyev *et al.*, 2020 ▸; Leatham *et al.*, 2023 ▸; Ahlers *et al.*, 2024 ▸), single-grid (Wen *et al.*, 2010 ▸; Morgan *et al.*, 2013 ▸) and speckle-based (Zdora, 2018 ▸; Berujon *et al.*, 2012 ▸) approaches, with synchrotron setups as summarized in Fig. 1 ▸. These newer setups directly resolve dark-field effects by measuring local blurring in the raw experimental image, creating a ‘family’ of dark-field approaches. The blurring is typically several micrometres wide, making these methods particularly well suited to high-resolution imaging, and hence complementary to grating interferometry and edge illumination.

To examine the similarities, differences and unique strengths of this subset of dark-field imaging approaches, developed largely by the authors, this work acquires dark-field data from two test samples and applies several relevant retrieval algorithms. Given that the different methods return different quantities for the dark-field signal due to differences in how scattering information manifests in the data and is handled during image retrieval, the current study is restricted to a qualitative comparison. Nevertheless, we believe that this qualitative comparison is an important step towards understanding how the dark-field signal varies across methods, serving as a foundation for future efforts to quantify the signal and relate it between different approaches. The remainder of this introduction provides an overview of these imaging setups and their associated dark-field retrieval algorithms, presented in the order of propagation-based, single-grid and speckle-based X-ray imaging.

### 1.1. Propagation-based X-ray imaging

Propagation-based imaging, shown in Fig. 1 ▸(*a*), requires no additional optics beyond what is required for conventional attenuation imaging, although it necessitates a relatively high degree of spatial coherence at the sample (Snigirev *et al.*, 1995 ▸; Cloetens *et al.*, 1996 ▸; Wilkins *et al.*, 1996 ▸). The distance between the sample and detector allows the coherent wavefield to self-interfere, resulting in bright and dark interference fringes that locally enhance the image contrast at edges/interfaces in the sample. Propagation-based imaging has been widely used for phase-contrast imaging (Gureyev *et al.*, 2009 ▸; Töpperwien *et al.*, 2018 ▸) and has only recently been employed to capture dark-field information (Gureyev *et al.*, 2020 ▸; Leatham *et al.*, 2023 ▸; Ahlers *et al.*, 2024 ▸).

In this setup, dark-field effects from porous or granular regions of a sample cause locally reduced image contrast (or blur) compared with what would be seen in the absence of these effects. This blur increases with decreasing X-ray energy (Ahlers *et al.*, 2024 ▸), as lower-energy X-rays are scattered to larger angles, and it also increases with increasing propagation distance between the sample and detector (Leatham *et al.*, 2023 ▸), as scattered X-rays spread over more pixels. These dependencies can be exploited to separate dark-field signals from attenuation and phase-contrast effects.

In this work, we use the dual-energy dark-field retrieval approach introduced by Ahlers and co-workers, which requires two propagation-based X-ray images acquired at different monochromatic energies (Ahlers *et al.*, 2024 ▸). We chose to explore this approach because it shows particular promise for dark-field imaging with photon-counting detectors, which are becoming increasingly attractive for routine clinical use (McCollough *et al.*, 2023 ▸). The approach of Ahlers and co-workers enables simultaneous retrieval of dark-field, attenuation and phase information from a single acquisition, with the two required images obtained via different energy bins in the detector (Ahlers *et al.*, 2025 ▸).

**Figure 2:**
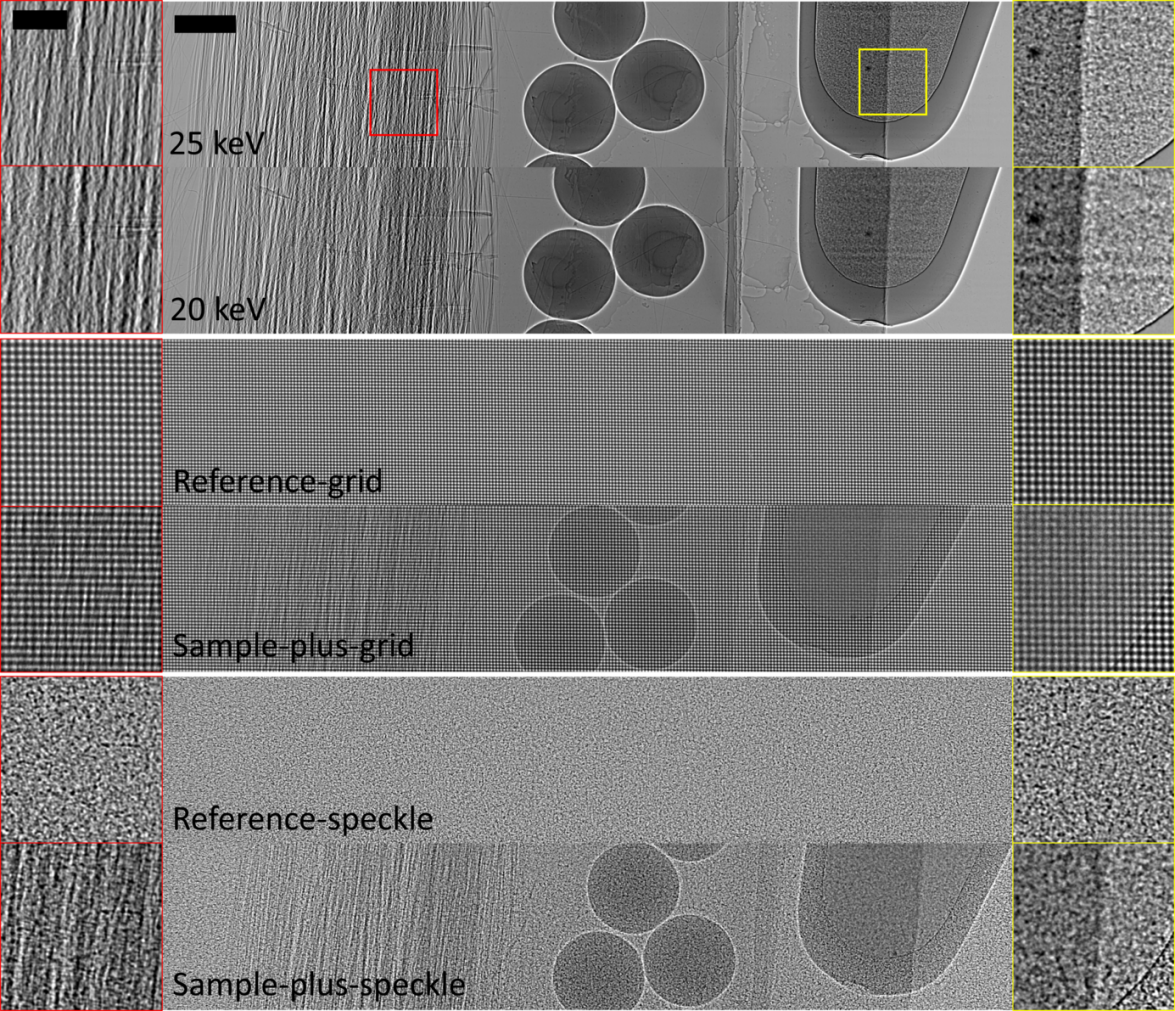
Images of the carbon-sphere sample, collected for the various dark-field approaches compared in this study. (Top) Dual-energy propagation-based approach. Dark-field contrast is generated by the difference in local blurring between two images collected at two sufficiently different X-ray energies (Ahlers *et al.*, 2024 ▸). Note that the horizontal features observed within the microsphere-filled tube at 20 keV originate from the flat-field correction. The illumination was not uniform, and the horizontal structures in it were blurred by the dark-field effects generated by the microspheres, preventing these variations from being fully divided out during the flat-field correction. This is analogous to the challenges of performing flat-field correction in the presence of strong phase effects (Homann *et al.*, 2015 ▸), but arises here due to dark-field effects. (Middle) The sample-induced dark-field effect in the single-grid approach blurs the 2D periodic reference-grid pattern (How & Morgan, 2022 ▸). (Bottom) In speckle-based imaging, dark-field contrast can be retrieved by tracking the speckle-blur between the reference-speckle and sample-plus-speckle images (Alloo *et al.*, 2023 ▸; Beltran *et al.*, 2023 ▸). The two black rectangles in the top left-hand images of this figure indicate the scale bars for the magnified and whole images (0.2 mm and 1 mm, respectively). Note that in the case of the multi-speckle approach, images are collected for multiple positions of the speckle generator, but these are not shown here.

Suitable data for the dual-energy dark-field approach are shown in Fig. 2 ▸(top), where propagation-based images of one of our test samples have been captured at energies of 20 keV and 25 keV. Dark-field effects are stronger in the lower-energy image, as indicated by the increased image blurring behind strongly scattering regions, which are highlighted in the red and yellow magnified regions within this figure.

The dual-energy propagation-based dark-field retrieval approach is derived from the Fokker–Planck equation for X-ray imaging, which models coherent (phase-shift) and diffusive (dark-field) flows of intensity in an X-ray imaging context (Paganin & Morgan, 2019 ▸; Morgan & Paganin, 2019 ▸). We employ the single-material local dark-field retrieval approach described by Ahlers and co-workers, where the first step is to use the X-ray Fokker–Planck equation to recover the sample thickness using both experimental images. The simultaneous consideration of phase and dark-field effects in this step means that the quality of the retrieved thickness is better than it would be if the blurring dark-field effects were ignored (Leatham *et al.*, 2024 ▸; Alloo & Morgan, 2025 ▸). The retrieved sample thickness is then used to simulate a so-called dark-field-free image, and the dark-field signal is then recovered by locally comparing the image visibility in the dark-field-free image with that in the original lower-energy experimental image [referred to as the ‘local’ method by Ahlers *et al.* (2024 ▸)]. The Fokker–Planck effective diffusion coefficient (*i.e.* the dark-field signal) is then calculated from the visibility using the propagation distance and period of local intensity variations.

This dark-field imaging approach is referred to as ‘self-referencing’ because it requires no external beam modulation. However, it does rely on the sample containing sufficient texture that the resulting image contrast can be visibly blurred at the lower imaging energy. If the sample lacks sufficient texture within an analysis window, the measured visibility is dominated by noise, which is then amplified by the ratio of visibilities. To address this, a small manually optimized regularization parameter can be introduced, which effectively suppresses this noise (see Table S1 in the supporting information for details).

### 1.2. Single-grid X-ray imaging

Single-grid imaging inserts a 2D-periodic modulator immediately before or after the sample, into the setup for propagation-based imaging [see Fig. 1 ▸(*b*) for the setup in which the modulator is upstream of the sample]. The modulation grid either attenuates (Wen *et al.*, 2010 ▸; Morgan *et al.*, 2011 ▸; How & Morgan, 2022 ▸) or phase-shifts (Morgan *et al.*, 2013 ▸; Rizzi *et al.*, 2013 ▸) the incident illumination to generate the periodic reference pattern, with the latter minimally attenuating the X-rays, thereby improving imaging speed. The modulation in the imaging illumination enables the detection of sample-induced gradients in the X-ray phase, increasing the sensitivity to subtle variations in the sample thickness or density relative to propagation-based imaging. While local shifts in the grid pattern reveal the phase of the X-ray wavefield, local blurring of the pattern reveals a dark-field effect.

The well defined periodic illumination in single-grid imaging enables controlled tuning of the system’s sensitivity to dark-field effects by adjusting the modulator-to-detector distance and/or the modulator, and hence the period of the generated reference pattern. This uniform reference pattern period allows quantitative analysis of the recovered dark-field signal, as the measured dark-field signal can be correlated with the number of structures generating that signal (Lim *et al.*, 2022 ▸; How & Morgan, 2022 ▸; How *et al.*, 2023 ▸). Periodic grids provide retrieved dark-field images with uniform spatial resolution and sensitivity across the entire field of view. This means that the imaging system is most sensitive to a single scattering signal, as determined by the grid period, while still having a certain sensitivity towards scattering signals of other length scales comparable with the grid period. Single-grid imaging is however vulnerable to Moiré artefacts if the sample contains periodic features similar in period to the grid pattern. Artefacts resembling the grid pattern may also arise in reconstructed images as a result of the varying sensitivity and the sampling process used for dark-field retrieval. In such cases, applying a post-processing median filter with a kernel size equal to the grid period, or an appropriate Fourier filter, can help suppress these artefacts.

To retrieve images using a single-grid setup, a reference-grid image and a sample-plus-grid image must be captured. The reference-grid image is captured with just the grid in the beam, and the sample-plus-grid image is captured when the sample is inserted into the grid-modulated beam. Fig. 2 ▸(middle) shows a pair of these images for one of the samples investigated in this work. In regions with dense unresolved microstructure, the grid pattern will be locally blurred or reduced in visibility, for example, behind the tube of microspheres shown in the yellow box in Fig. 2 ▸(middle). A sample with unresolved fibres or orientated structures will predominantly blur the grid pattern in the direction perpendicular to those fibres, as seen in the red box in Fig. 2 ▸(middle), revealing a directional dark-field signal (Pavlov *et al.*, 2021 ▸; Croughan *et al.*, 2023 ▸).

In this study, we apply the single-exposure dark-field retrieval approach developed by How & Morgan (2022 ▸) to track mathematically how the reference grid pattern is modified in the presence of a sample. To retrieve the dark-field, the approach uses local pixel-wise cross-correlation analysis to quantify changes in visibility between the reference and sample-plus-grid images. Specifically, the periodic grid intensity patterns are modelled as a sinusoid, and the cross-correlation is performed within local analysis windows (typically sized to match the grid period) in both the reference-grid and sample-plus-grid images to measure the differences in this model with and without the sample present. The explicit modelling and tracking of intensity changes imposed by the sample means that phase-gradient and dark-field effects are simultaneously considered and thus decoupled, within limitations imposed by the choice of analysis window size. Readers are referred to How and Morgan’s paper for specific details regarding the derivation and implementation of the algorithm (How & Morgan, 2022 ▸), and to the work of Croughan *et al.* (2023 ▸) for a directional dark-field generalization of the algorithm.

The retrieved dark-field image from How and Morgan’s single-grid approach represents a visibility reduction of the grid pattern, which is a fundamentally different quantity to the Fokker–Planck effective diffusion coefficient retrieved via the dual-energy approach of Ahlers *et al.* (2024 ▸), and also by the two speckle approaches we describe in the next sub-section. To facilitate a closer comparison with the other dark-field images shown in this work, we convert the single-grid-retrieved visibility reduction *V* to a Fokker–Planck effective diffusion coefficient *D* using the relationship *D* = −(*p*^2^/4π^2^Δ^2^)ln(*V*) (Morgan & Paganin, 2019 ▸; Paganin *et al.*, 2023 ▸), where *p* is the period of the grid pattern and Δ is the distance between the sample and detector. Note that all dark-field images presented in this manuscript represent the Fokker–Planck-defined diffusion coefficient *D*, a dimensionless quantity that is directly related to the opening angle of the local diffuse scattering fan. Larger *D* values in an image correspond to more diffuse scattering, or greater dark-field effectiveness, within the pixel.

### 1.3. Speckle-based X-ray imaging

Speckle-based X-ray imaging, shown in Fig. 1 ▸(*c*), replaces the periodic modulator in single-grid imaging with a spatially random one. This inherently alleviates the need for high precision in the mask fabrication, to the extent that a simple sheet of sandpaper (Morgan *et al.*, 2012 ▸) or a biological membrane (Berujon *et al.*, 2012 ▸) can be used as a modulator. A reference-speckle and sample-plus-speckle image pair must be recorded to capture and then retrieve the dark-field signal; an example data pair is shown in Fig. 2 ▸(bottom). Similar to the case of single-grid imaging, dark-field effects manifest as speckle blurring or a reduction in visibility in the sample-plus-speckle image compared with the reference-speckle image. Some dark-field retrieval approaches for speckle-based imaging require a single pair of data, while others require multiple pairs acquired by moving the modulator to different transverse positions—either equidistant or random, depending on the specific retrieval algorithm—and collecting a reference-speckle and sample-plus-speckle image pair at each step (Zdora, 2018 ▸). The use of multiple data pairs can improve the spatial resolution of the retrieved images compared with those obtained from a single dataset, as each pair encodes slightly different information about how the sample modifies the incident X-rays. Additionally, the increased diversity of information from data acquired at different mask positions improves the conditioning of the ill-posed inverse problem of image retrieval, generally yielding images with higher fidelity and reduced noise sensitivity.

Given the random nature of the reference-speckle pattern in terms of the size and visibility of speckles, the sensitivity across the system’s field of view can vary. For example, speckle patterns with regions containing large speckles exhibit reduced sensitivity; large speckles are more difficult to shift (phase effects) or blur (dark-field effects) than smaller ones, so changes induced by the sample are subtle and can result in the image-retrieval algorithm experiencing difficulties in these regions. This issue can be somewhat alleviated in multiple-pair speckle-based imaging, since a smaller speckle is likely to pass over a given area in a subsequent image pair that will then contribute to the final reconstruction. The variation in speckle size in speckle-based imaging can be advantageous for samples with hierarchical structures, enabling sensitivity to a broad range of dark-field scattering signals corresponding to the range of characteristic length scales in the speckle pattern (Meyer *et al.*, 2021 ▸). This effect has been demonstrated by Lindberg *et al.* (2025 ▸) and Magnin *et al.* (2025 ▸), who explored retrieved phase and dark-field images for masks with different speckle sizes and visibilities.

In this work, we explore two dark-field algorithms for speckle-based imaging: one that requires a single pair of images (Beltran *et al.*, 2023 ▸) (referred to as single-speckle imaging hereafter) and one that requires multiple pairs (Alloo *et al.*, 2023 ▸) (referred to as multi-speckle imaging hereafter). Like the dual-energy propagation-based dark-field retrieval method described earlier, both of the speckle-based dark-field retrieval algorithms we explore are derived from the X-ray Fokker–Planck equation. However, dissimilarly, these speckle-based algorithms are ‘global’ as the dark-field image is retrieved by evaluating an analytically derived equation based on the relevant Fokker–Planck formalism using entire experimental images. The Fokker–Planck algorithms we explore also differ in their underlying assumptions, which are designed to simplify the mathematics of solving the associated Fokker–Planck equation. An important assumption that varies across these algorithms is the restriction on sample composition: the dual-energy algorithm assumes the sample is a single material, requiring *a priori* information of both components δ and β of a sample’s complex refractive index decrement, where *n* = 1 − δ + *i*β is the full refractive index, whereas the two algorithms for speckle-based imaging assume the sample is homogeneous, meaning that γ = δ/β is constant, requiring *a priori* information of this γ value for successful dark-field retrieval. The assumption of constant γ allows algorithms for speckle imaging to be applied to multi-material samples, in which constituent materials may have different values for their δ and β parameters, but their δ/β ratio is approximately the same across all materials. In contrast, the single-material assumption in the dual-energy propagation-based approach of Ahlers *et al.* may be less reliable when the sample contains materials with significantly different attenuation and refraction characteristics, as it assumes δ and β are individually constant throughout the sample. It is worth noting that How and Morgan’s dark-field algorithm for single-grid imaging does not require any *a priori* information, as the dark-field signal is retrieved entirely from local pixel-wise measurements within the acquired data.

In addition to the homogeneous-sample assumption described above, the two speckle-based dark-field retrieval algorithms we investigate make fundamentally different assumptions. The single-speckle dark-field imaging algorithm of Beltran *et al.* consists of two steps: (i) the sample’s phase is approximated using a conventional transport-of-intensity-based phase retrieval (Paganin *et al.*, 2002 ▸) and (ii) the dark-field signal is retrieved by substituting the approximated phase into the Fokker–Planck equation and rearranging. The first phase-retrieval step assumes that dark-field effects are weak compared with attenuation and propagation-based phase effects, allowing a sample-only image to be estimated by dividing the sample-plus-speckle image by the reference-speckle image. This assumption is reasonable if the reference speckles are not significantly blurred or shifted by the sample. This can be ensured by properly adjusting the imaging setup before acquisition and by comparing the reference-speckle and sample-plus-speckle images to verify that the speckles are only moderately distorted, that is, not saturated, lost or shifted by several pixels. The two-step nature of the method of Beltran *et al.*, in which dark-field effects are neglected in the first step, means that phase and dark-field effects are not considered simultaneously. However, as Beltran *et al.* suggested, the initial phase approximation can be updated to account for dark-field effects using an iterative extension of the method, which we do not implement in this work.

If several imaging acquisitions of the sample are possible, pairs of reference-speckle and sample-plus-speckle images can be captured, and a multi-exposure dark-field retrieval algorithm can improve the quality of the retrieved images. Here, we explore the multi-speckle dark-field approach of Alloo *et al.* (2023 ▸), the most generalized variant of the Multimodal Intrinsic Speckle-Tracking (MIST) algorithm (Pavlov *et al.*, 2020 ▸). By using multiple data pairs for image retrieval, the approach simultaneously retrieves phase and dark-field images, using the additional data to disentangle dark-field and phase-contrast effects. Specifically, it requires a minimum of four speckle imaging pairs, each of which establishes a lin­ear­ized form of the Fokker–Planck equation for speckle imaging containing four unknown variables corresponding to the desired reconstructed images. The generated system of linear equations can then be solved using standard methods; Alloo *et al.* suggest using a Tikhonov-regularized QR decomposition. The phase and dark-field images are then computed using Fourier-space filtering to give a suitable combination of solutions from the Tikhonov-regularized QR decomposition. Additional data above the minimum of four speckle-imaging pairs can further improve image quality, as the linear system to be solved becomes overdetermined, thereby increasing redundancy in the data.

### 1.4. Focus and scope

In this paper, we aim to explore qualitatively the similarities and differences within this family of X-ray dark-field methods by applying the described approaches to a common set of samples imaged at a single facility. We restrict the scope of this study to these four emerging ‘direct-resolved dark-field’ methods, for which we have expertise and available experimental setups on the MicroCT beamline at the Australian Synchrotron. Comparisons with those X-ray dark-field imaging techniques that are typically applied at larger length scales, such as grating interferometry and edge-illumination, are left for future studies. We also restrict the scope to one dark-field retrieval algorithm for each experimental imaging method, and for those methods where more than one algorithm has been developed, we refer the reader to previous studies that focus on retrieval algorithm comparisons for a given method, for example different speckle-tracking algorithms on the same speckle-based imaging data (Pavlov *et al.*, 2020 ▸; Rouge-Labriet *et al.*, 2021 ▸; Zandarco *et al.*, 2024 ▸; Celestre *et al.*, 2025 ▸; Lindberg *et al.*, 2025 ▸). Regarding how various grid/speckle reference patterns influence the quality of retrieved dark-field images, we also refer the reader to previous studies on this topic (How & Morgan, 2022 ▸; Beltran *et al.*, 2023 ▸; Magnin *et al.*, 2025 ▸). Note that the datasets for the single-grid and single-speckle algorithms in this paper can be exchanged, with the results already shown in the corresponding papers (How & Morgan, 2022 ▸; Beltran *et al.*, 2023 ▸).

Our choice of approach ensures that (i) the experimental imaging data are acquired with consistent experimental parameters, *i.e.* the same coherence, source size, X-ray energy, pixel size, exposure time and detector, and (ii) the dark-field retrieval algorithms are implemented by the authors of each method, using optimal input parameters and settings to ensure that the retrieved images are of the highest possible quality for each dataset.

The remainder of this paper is organized as follows. Section 2 describes the experimental data collection procedures for the X-ray dark-field imaging techniques examined in this work: propagation-based, single-grid and speckle-based X-ray imaging. Section 3 presents the retrieved dark-field images obtained using four different retrieval algorithms (one for propagation-based, one for single-grid and two for speckle-based imaging), highlighting shared features and distinctive characteristics. Finally, we summarize our findings and provide a user-oriented table comparing the dark-field imaging setups and retrieval algorithms explored within this article.

## 2. Experimental methods

We imaged two test samples using three X-ray dark-field imaging techniques that can be implemented on the Australian Synchrotron’s MicroCT beamline. As is typical for technique-development studies, the test samples were constructed from materials with well defined structures and hence known X-ray properties, namely attenuation, phase-shift and dark-field effects. It is worth noting that all dark-field-generating mater­ials imaged in this study induce relatively strong dark-field signals, *i.e.* they produce visible-by-eye image blurring in the experimental data, as shown in Fig. 2 ▸. Materials that generate only weak dark-field signals, for example, biological tissue, which may produce image blurring too subtle to be seen by eye, were not considered here and are left for future work. The test samples, which we will refer to as the ‘carbon-sphere’ sample and the ‘four-rod’ sample, are shown in Fig. 1 ▸(*d*).

Imaging was performed in the first experimental hutch of the MicroCT beamline at the Australian Synchrotron, which is located 24 m from the bending-magnet source. The detector system for all techniques was a pco.edge 5.5 complementary metal-oxide-semiconductor (CMOS) camera, which had 2560 × 2160 pixels with 6.5 µm square pixels, coupled to a GGG:Eu/Tb scintillator with a 1× optical lens placed in between. For each image type (*e.g.* reference image, sample image *etc.*) across all dark-field imaging setups, 30 images were captured with an exposure time of 0.04 s. These were then averaged and used in the corresponding dark-field image retrieval algorithm. Consequently, when a particular dark-field retrieval method required multiple imaging datasets, a higher total X-ray exposure of the sample and hence imaging time was necessary. For the dual-energy propagation-based approach, images at monochromatic X-ray energies of 20 keV and 25 keV were acquired with a fixed propagation distance of 0.7 m between the sample and the detector. For single-grid and speckle-based imaging, the wavefront modulator was positioned 0.3 m in front of the sample, the sample-to-detector propagation distance was 0.7 m and a 25 keV monochromatic X-ray beam was used.

A geological stainless steel sieve with 25 µm square apertures and 27 µm thick wires, producing a 52 µm period grid pattern at the detector, was used for single-grid imaging. For speckle-based imaging, two layers of P800 grit sandpaper were inserted in place of the grid. The generated speckle pattern at the detector had an average speckle size of 18.3 µm, measured by taking the full-width at half-maximum of the reference-speckle pattern’s autocorrelation function (Goodman, 2020 ▸). To capture multi-speckle imaging data from both samples, the speckle mask was moved to 13 random transverse positions, collecting 13 pairs of reference-speckle and sample-plus-speckle images. For all imaging methods, dark-current and flat-field images were acquired to correct for detector electronic noise and X-ray beam inhomogeneities. The imaging data for the carbon sphere using each of the setups can be downloaded from an open Zenodo repository (Alloo, 2025 ▸).

## 3. Results and discussion

Dark-field images of the carbon-sphere and four-rod samples were retrieved using the retrieval algorithms described in Section 1. For the propagation-based data, the dual-energy approach of Ahlers *et al.* (2024 ▸) was applied; for the single-grid data, How and Morgan’s single-exposure retrieval method was used (How & Morgan, 2022 ▸); for the speckle-based data, we applied both the single-exposure approach of Beltran *et al.* (2023 ▸) and the multi-exposure approach of Alloo *et al.* (2023 ▸). In each case, an author of the respective method performed the image processing, ensuring the correct tuning of all required input parameters. A description of these input parameters for both samples is provided in Table S1 of the supporting information.

Although our focus is on dark-field retrieval, transmission images (phase-retrieved in relevant cases) were also obtained using all image-retrieval algorithms. These transmission images, along with a brief description, are provided in the supporting information. The Python scripts used to implement each algorithm are available from the primary author of the respective method upon request.

In this section, we present the retrieved dark-field images obtained using the various methods explored, and discuss their similarities and differences based on visual inspection and qualitative analysis. Quantitative comparisons are left for a future study for two reasons: dark-field quantification has not yet been developed for all the techniques, and the samples were chosen to provide natural variations in texture and thickness, and not to provide areas of uniform dark-field signal. A quantitative comparison would be a large study in itself, for example requiring a range of microstructure sizes to test the range of sensitivity of each technique.

The study presented here aims to simplify comparisons by fixing the parameters that influence imaging system quality measures such as sensitivity, spatial resolution and signal-to-noise ratio. This is achieved by using the same X-ray source and detector throughout, and the same energy, except for the dual-energy method. The sensitivity of these ‘directly resolved’ techniques to weak dark-field signals will depend upon the visibility of the reference pattern (45.5% for the grid versus 32.3% for the speckle versus 10.5% for the sample’s own texture) and the pixel size (Morgan *et al.*, 2013 ▸), which is fixed. The spatial resolution of the retrieved images will depend on the period of the reference patterns (eight pixels for the grid versus three pixels for the speckle), the spatial resolution of the imaging system, which is fixed, and the method of retrieval, as discussed below. The signal-to-noise ratio will depend on the exposure time, which is fixed, the number of exposures, which varies, and the retrieval method. The observed qualitative differences in the retrieved dark-field images will therefore be primarily a result of the assumptions made by the retrieval method, the analytical approach used and the minimized unavoidable differences in setup/method noted above (*e.g.* sampling period, reference visibility). By controlling the setup and samples as much as possible, we can determine in which cases reconstruction artefacts and edge signals are likely to appear.

The experiment, analysis process and resulting images enabled a practical user guide to be assembled. This is presented at the end of the section and can assist users in selecting the most suitable method from among those investigated for a given experimental setup, sample type and imaging aim. To support transparency, all retrieved images from the various approaches have been uploaded to the previously mentioned open Zenodo repository (Alloo, 2025 ▸), enabling readers to inspect visually the differences and similarities discussed in this section.

**Figure 3:**
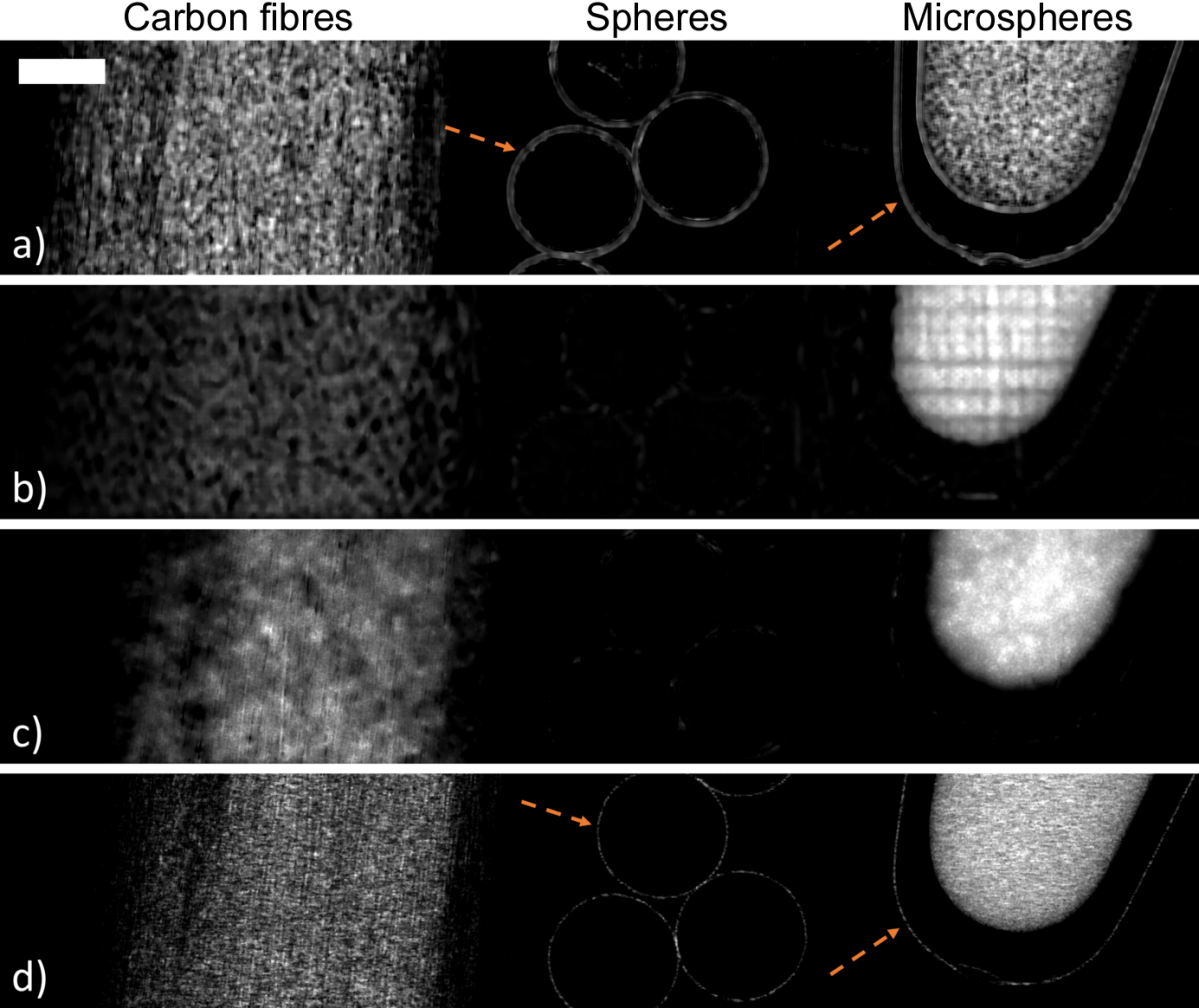
Retrieved dark-field images of the carbon-sphere sample using the different retrieval approaches: (*a*) dual-energy propagation-based, (*b*) single-grid, (*c*) single-speckle and (*d*) multi-speckle. Dashed orange arrows in panels (*a*) and (*d*) highlight edges in the sample that are only resolved by the corresponding approaches. The white rectangle in panel (*a*) denotes the 1 mm scale bar for all images. The grayscale ranges for the images are [min (black), max (white)]: (*a*) = [0.0, 1.5] × 10^−10^, (*b*) = [0.0, 1.0] × 10^−10^, (*c*) = [0.0, 4.0] × 10^−11^ and (*d*) = [0.0, 6.0] × 10^−11^.

**Figure 4:**
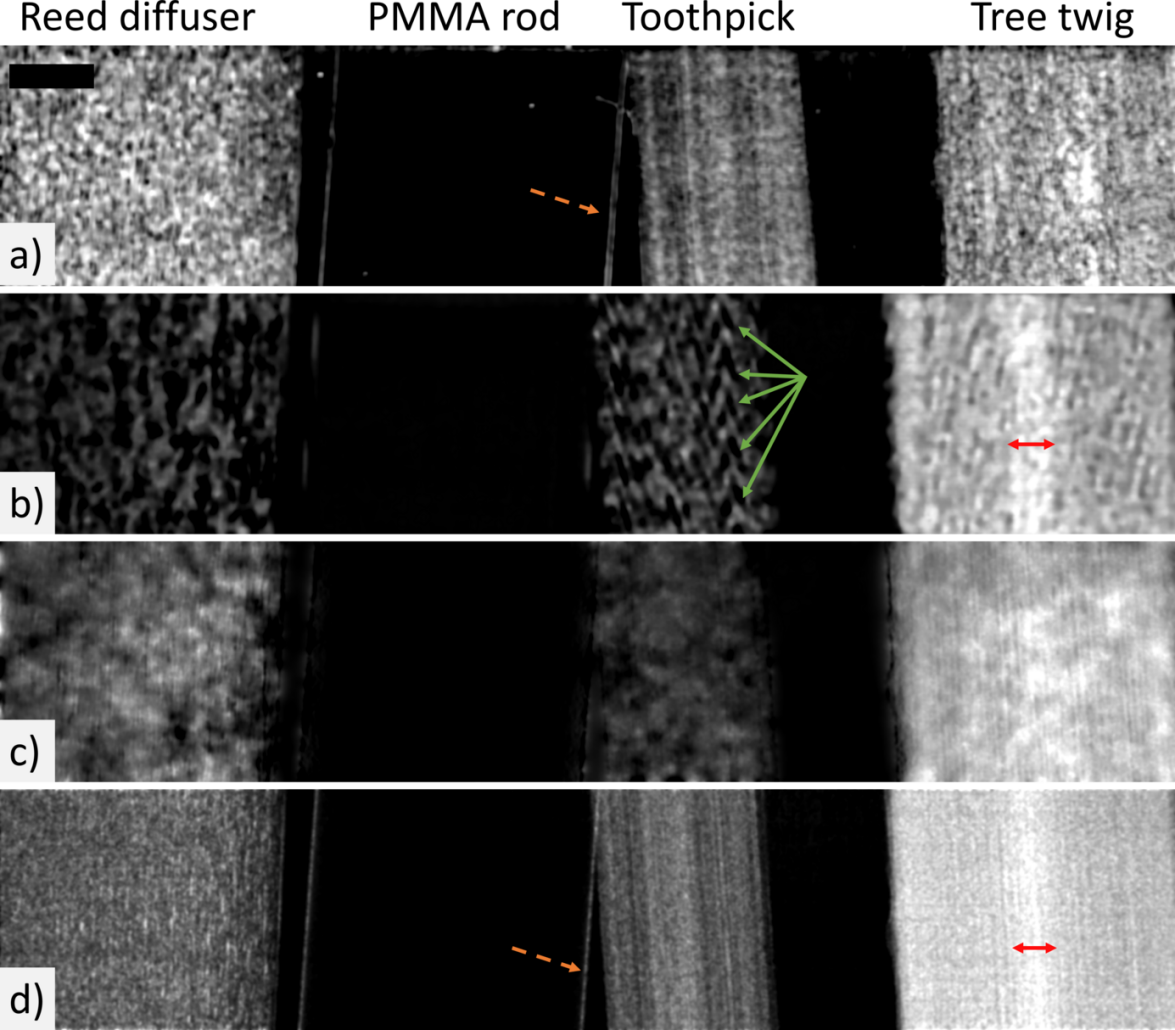
Retrieved dark-field images of the four-rod sample using the different retrieval approaches, with a two-pixel standard deviation Gaussian filter applied post-retrieval: (*a*) dual-energy propagation-based, (*b*) single-grid, (*c*) single-speckle and (*d*) multi-speckle. Dashed orange arrows in panels (*a*) and (*d*) highlight edges in the sample that are only resolved by the corresponding approaches. Solid green arrows in panel (*b*) indicate Moiré-like artefacts arising from the toothpick fibres, generating phase-contrast fringes comparable in size to the period of the imaging grid. Red arrows in panels (*b*) and (*d*) span the tree twig’s central pith region, a feature visible only with these two approaches. The black rectangle in panel (*a*) denotes the 1 mm scale bar for all images. The grayscale ranges for the images are [min (black), max (white)]: (*a*) = [0.0, 1.2] × 10^−10^, (*b*) = [0.0, 8.9] × 10^−11^, (*c*) = [0.0, 4.1] × 10^−11^ and (*d*) = [0.0, 3.9] × 10^−11^.

The retrieved dark-field images of the carbon-sphere and four-rod samples are shown in Figs. 3 ▸ and 4 ▸, respectively. At a coarse-grained level where a sufficient degree of spatial smoothing is applied, the retrieved dark-field images of each sample appear relatively similar, with each method clearly differentiating regions that generate dark-field signal from those regions that do not. For the carbon-sphere sample, the bundle of carbon fibres and the tube of PMMA microspheres generate a dark-field signal, as shown in Fig. 3 ▸. The large spheres, which are the most prominent feature in the raw or transmission images, do not generate a bulk dark-field signal. For the four-rod sample, those objects with underlying microfibre structures generate a dark-field signal, with the reed diffuser, toothpick and tree twig all visible in all the dark-field images in Fig. 4 ▸, and the highly attenuating solid PMMA rod disappears.

As all approaches retrieve measurable dark-field effects from sub-resolution structures, they can be considered to be generally consistent with each other. Although generally consistent, there are some subtle differences between the dark-field images retrieved using the different approaches. The first is the sensitivity of each approach to edge-related dark-field signal (Born & Wolf, 1999 ▸; Borghi, 2015 ▸; Yashiro & Momose, 2015 ▸; Alloo *et al.*, 2025 ▸). Both the dual-energy and multi-speckle approaches are more sensitive to edge-related contributions in the dark-field signal, with sharp edges in both samples visible in these images, as indicated by the orange dashed arrows in Figs. 3 ▸ and 4 ▸. The edges in the dual-energy-retrieved dark-field images are less sharply defined than those in the multi-speckle results, spanning approximately 20 and 5 pixels, respectively. This difference may reflect the modulation and sampling strategies used in these imaging methods. The dual-energy approach uses only two sample exposures and applies a window (10 × 10 pixels in this case) to measure local visibility and hence perform image retrieval. In contrast, the multi-speckle images presented here are reconstructed from 13 exposures, where the deliberately introduced modulation provides trackable features in the beam, enabling information recovery even at sharp boundaries that may otherwise be challenging to resolve.

Another difference is the presence of artefacts in the dark-field images retrieved from a single set of imaging data. Imaging methods that rely on a single exposure are generally more susceptible to artefacts arising from either imperfections in the reference pattern or limitations in the image-retrieval process, as data redundancy is minimal, and it is likely that this is what is being observed in our images. Specifically, the single-grid-retrieved images exhibit remnant grid artefacts and/or horizontal illumination structures within the carbon-sphere sample’s tube of microspheres [Fig. 3 ▸(*b*)], missing signal in the four-rod sample’s reed diffuser, and Moiré-like artefacts in the toothpick of the four-rod sample [Fig. 4 ▸(*b*)]. Single-grid imaging is also susceptible to Moiré artefacts—periodic patterns indicated by the green arrows in Fig. 4 ▸(*b*)—which arise when propagation-based phase-contrast fringes in the data are similar in size and contrast to the grid pattern, such as the fringes from the wood fibre bundles in the toothpick. In regimes where the grid pattern is better separated from the sample contrast (*e.g.* larger samples), this issue is avoided (How *et al.*, 2026 ▸).

For both samples, the single-speckle-retrieved dark-field images [Figs. 3 ▸(*c*) and 4 ▸(*c*)] exhibit a ‘splotchy’ (*i.e.* irregular and speckled) signal across the entire image. This characteristic may be attributed to the fact that the sampling efficacy of this approach is strongly influenced by speckle pattern characteristics such as speckle size, visibility and sparsity, with improved performance seen with high-visibility periodic grids (Beltran *et al.*, 2023 ▸). While the single-exposure dark-field retrieval approaches generally exhibit lower image quality than approaches using multiple data sets, they offer the important advantage of enabling dynamic imaging (Croughan *et al.*, 2023 ▸; Smith *et al.*, 2024 ▸; How *et al.*, 2026 ▸), which is not currently feasible with multi-exposure techniques [except in the case of repeated motion (Gradl *et al.*, 2018 ▸)]. The dual-energy propagation-based approach is also capable of dynamic imaging if a dual-channel photon-counting detector is used, as the two required data sets can then be captured simultaneously (Ahlers *et al.*, 2025 ▸).

A further notable difference between the dark-field images across the approaches occurs at the centre of the tree twig in the four-rod sample. The tree twig was composed of two distinct types of wood, an outer cortex and an inner pith, each with different structural characteristics that are visible to the naked eye. Only the single-grid and multi-speckle dark-field images clearly distinguish between these two regions, with the inner pith marked with a red arrow in Figs. 4 ▸(*b*) and 4 ▸(*d*). This feature is perhaps faintly visible in the dual-energy-retrieved dark-field image [Fig. 4 ▸(*a*)] but is partially obscured by textured structures resembling propagation-based phase-contrast fringes. It is not easily identified in the single-speckle result [Fig. 4 ▸(*c*)], probably because this retrieval method is highly sensitive to the characteristics of the reference speckle pattern; in this case, bright speckles over the central region of the twig mask the feature.

The dark-field approaches also vary in how they represent the relative signal strength across materials within a sample. This effect is evident for both the carbon-sphere and four-rod samples (Figs. 3 ▸ and 4 ▸, respectively) where the dual-energy retrieval produces comparable dark-field strengths across different dark-field-generating materials, while the other approaches measure a distinct difference in dark-field signal strength between, for example, the carbon fibres and the microspheres. This could be due to a change in the X-ray energy/energies used for the reconstruction, since the strength of the dark-field signal for a given microstructure size will change with energy (Ahlers *et al.*, 2024 ▸), and this method uses 20 keV and 25 keV measurements, while the other methods use only 25 keV. Future work could quantify the dark-field dependencies on energy, reference feature size and sample-to-detector propagation distance, as well as microstructure size, material and packing density for each method, which would help in identifying the optimal imaging modality for a given application.

**Figure 5:**
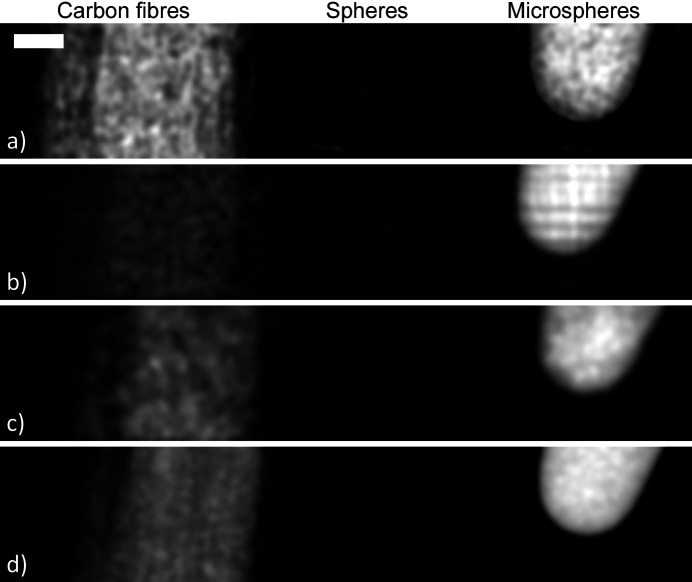
The retrieved dark-field images shown in Fig. 3 ▸ (with the same labelling of panels) with a ten-pixel standard deviation Gaussian filter applied. The approaches used to retrieve each of the images were: (*a*) dual-energy propagation-based, (*b*) single-grid, (*c*) single-speckle and (*d*) multi-speckle. The white rectangle in panel (*a*) denotes the 1 mm scale bar for all images. The grayscale ranges for the images are [min (black), max (white)]: (*a*) = [0.0, 9.7] × 10^−11^, (*b*) = [0.0, 9.1] × 10^−11^, (*c*) = [0.0, 4.7] × 10^−11^ and (*d*) = [0.0, 4.0] × 10^−11^.

Despite differences in the retrieved dark-field images across the approaches explored, our results demonstrate that all methods produce generally consistent solutions; that is, structures generating dark-field effects can be mapped, albeit to varying degrees, using any of the approaches. To highlight this consistency further, particularly in the slowly varying low-spatial-frequency components of the dark-field signal, a Gaussian filter with a standard deviation of ten pixels was applied to all dark-field images of the carbon-sphere sample. The resulting images are shown in Fig. 5 ▸. The Gaussian filter is a low-pass filter, suppressing high-frequency components to emphasize that the large-scale structures seen in the dark-field images are consistently retrieved by all approaches, for example the bundle of carbon fibres and the tube of microspheres. The fine-scale differences observed between the retrieved dark-field images arise from artefacts or method-dependent retrieval of edges. Thus, we apply a Gaussian filter to the images in Fig. 5 ▸ to allow a comparison that emphasizes the consistent low-spatial-frequency information, allowing a direct assessment of how consistently the different methods recover relative dark-field intensities across the various sample materials, independent of method-specific high-frequency artefacts and noise. This processing step demonstrates the agreement between the methods for features producing low-spatial-frequency dark-field signals (*i.e.* regions with dense microstructure that are uniformly textured) and highlights that differences occur mainly in the high-spatial-frequency components.

**Figure 6:**
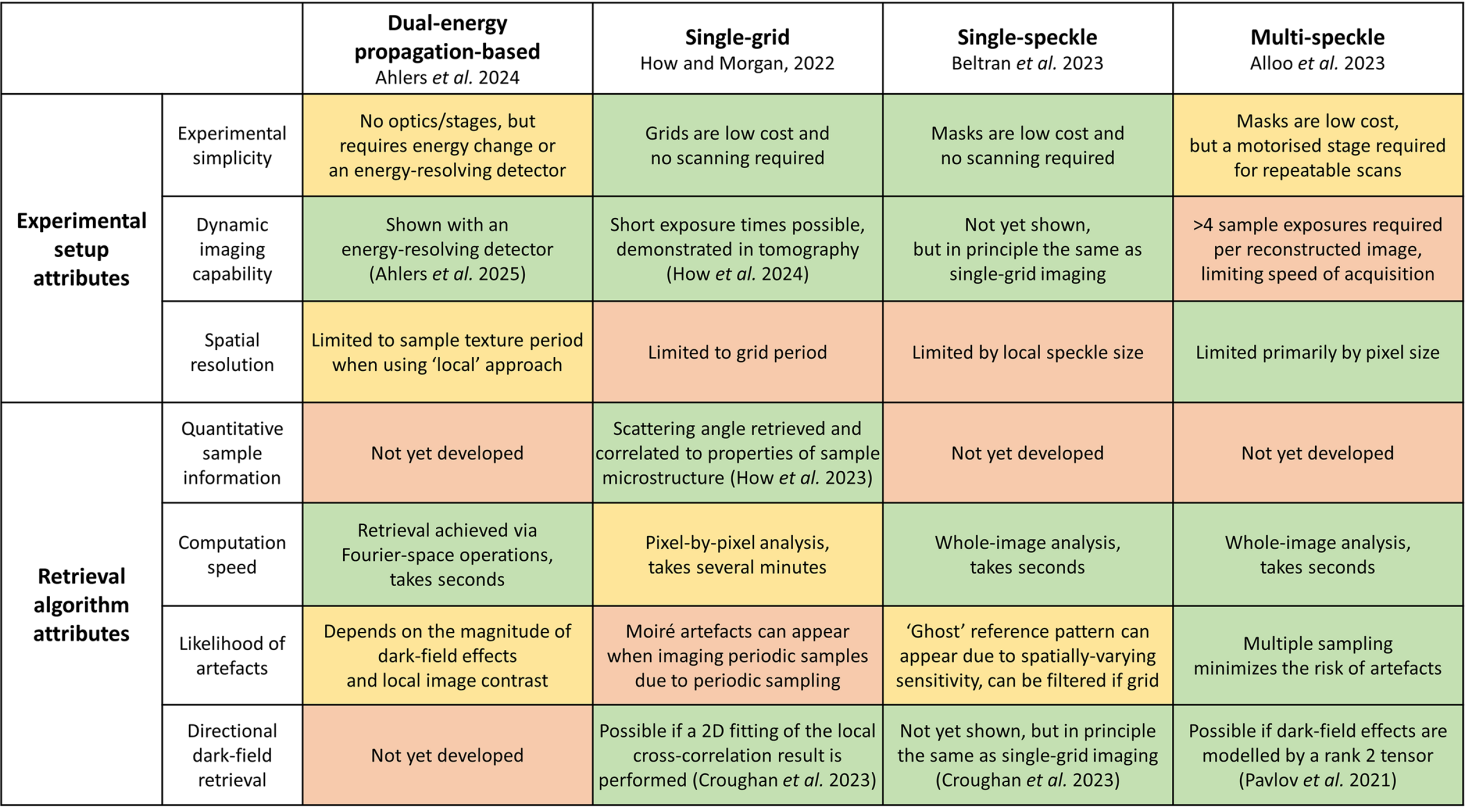
Summary of the strengths and limitations of each dark-field-retrieval approach (top row), assessed against the desirable attributes listed down the left-hand column.

The demonstrated agreement among the dark-field imaging approaches explored in this study reveals an important result: if one wishes to image an object and visualize regions of dense microstructure, such as microspheres or fibre bundles, any of the approaches—dual-energy, single-grid, single-speckle or multi-speckle—can be employed. Depending on the specific imaging requirements, such as capturing a dynamic process, detecting high-resolution structural detail or minimizing radiation exposure, users can select the most appropriate technique to meet their needs. To assist with this selection, we summarize in Fig. 6 ▸ the strengths and limitations of the investigated dark-field approaches, based on lessons from the experiments, the analysis experience, the results presented here, our collective expertise with these techniques, and the wider literature. The dark-field approaches are listed along the top of the table, and desirable attributes relating to both the experimental setup and the retrieval algorithm are listed down the left-hand column. Each cell is colour-coded (green, orange or red) to indicate how well a given approach satisfies the specified attribute, where green denotes the best performance and red the weakest. This table serves as a practical guide for dark-field imaging users, helping them select the most suitable approach (out of the four particular methods compared in the present study) for their specific experimental constraints. It is important to note that ongoing developments across most methods mean that this table will probably evolve in the coming years. For clarity, we distinguish between experimental advantages and algorithmic advantages, as these correspond to two distinct stages of the dark-field imaging process: first, choosing an appropriate imaging setup, and second, selecting a suitable retrieval algorithm for accurate reconstruction.

## 4. Conclusion

We have compared a subset, or ‘family’, of X-ray dark-field imaging approaches that extract the dark-field signal by directly resolving the image blur it induces. Our aim was to clarify how these different approaches capture and reconstruct the dark-field signal from the same material. Imaging data were acquired from two samples using propagation-based, single-grid and speckle-based setups, and the corresponding algorithms—each of which we have expertise in implementing—were used to recover dark-field images.

We found that all of the investigated approaches recover the primary (low-spatial-frequency) contribution to dark-field contrast in regions where it is expected. However, discrepancies emerged at higher spatial frequencies (*e.g.* at edges) and in terms of potential reconstruction artefacts. These differences are somewhat unsurprising, as each setup encodes dark-field information differently in the experimental data, and the retrieval algorithms vary in how they process and interpret that information.

Having shown that all these methods capture the primary component of the dark-field signal, we have discussed how users can select a method best suited to their specific imaging requirements. To support this, we provide a summary table (Fig. 6 ▸) to guide users in choosing an appropriate dark-field technique (out of the four particular methods compared in this paper) for their imaging goals.

We hope this work contributes to a broader discourse on the complementarity of dark-field imaging approaches, highlighting that each offers distinct strengths and limitations suited to different applications. Future studies could focus on quantifying the dark-field signal across different experimental setups and retrieval algorithms, which remains an open question in dark-field imaging research.

## 5. Related literature

For further literature related to the supporting information, see Hubbell & Seltzer (1995 ▸).

## Supplementary Material

Supporting information. DOI: 10.1107/S1600577525011403/vl5049sup1.pdf
