# Supplementary material for: Demonstration of a family of X-ray dark-field retrieval approaches on a common set of samples
Source: J Synchrotron Radiat. 2026 Jan 22;33(Pt 2):437–47. doi: 10.1107/S1600577525011403 (PMC12948023; doi:10.1107/S1600577525011403)
Supplement: Supplementary file 1 [file s-33-00437-sup1.pdf]

JOURNAL OF SYNCHROTRON RADIATION

Volume 33 (2026)

Supporting information for article:

Demonstration of a family of X-ray dark-field retrieval approaches on a common set of samples

Samantha J. Alloo, Ying Ying How, Jannis N. Ahlers, David M. Paganin, Michelle K. Croughan and Kaye S. Morgan

Supplementary material for S. J. Alloo *et al.*, 'Demonstration of a family of X-ray dark-field retrieval approaches on a common set of samples'

November 16, 2025

1 Image retrieval parameters

Table 1 lists the parameters employed by each image retrieval algorithm to reconstruct the dark-field images presented in the main manuscript and the corresponding transmission images shown in Figs. 1 and 2 below.

2 Retrieved transmission images

As mentioned in the main manuscript, a transmission image is retrieved alongside the dark-field image for the dark-field approaches we explored. The retrieved transmission images for the carbon-sphere and four-rod samples using the different approaches are shown below in Figs. 1 and 2, respectively. Importantly, each approach is based on different assumptions regarding the effects contributing to the retrieved transmission, and therefore, the retrieved images differ accordingly.

The approaches based on the Fokker–Planck equation—namely the dual-energy, single-speckle, and multi-speckle methods—couple phase and attenuation via the projection approximation under the assumption of a homogeneous sample with a constant δ/β ratio. Due to this shared assumption across these approaches, they yield similar transmission images that correspond to the acquired propagation-based phase-contrast images, but with the Fresnel fringes at edges corrected for and exploited to enhance the quality of the retrieved transmission image. Because the phase-contrast fringes are effectively removed in these cases, we often refer to the resulting images as phase-retrieved transmission images. Panels a), c), and d) of Figs. 1 and 2 show the phase-retrieved transmission images using the dual-energy, single-speckle, and multi-speckle approaches for the carbon-sphere and four-rod samples, respectively. In contrast, the single-grid method simultaneously retrieves transmission, differential phase-contrast, and dark-field images. For both samples studied, the differential phase-contrast images were noisy and contained no useful information; therefore, they are not shown. Since the single-grid method does not account for propagation-based phase contrast, fringes remain visible at material interfaces in its retrieved transmission images, as shown in Figs. 1b and 2b, for the carbon-sphere and four-rod sample, respectively.

Table 1: Summary of the parameters used with each retrieval algorithm in the reconstruction of the carbon-sphere and four-rod sample dark-field images.

**Algorithm**	**Retrieval parameters**
Dual-energy-propagation-based [1]	The approach assumes that both samples are composed of a single material: polymethyl methacrylate (PMMA). The required *a priori* complex refractive index parameters were taken as the theoretical δ and β values at 20 keV and 25 keV: $\delta_{\text{PMMA; 20 keV}} = 6.663 \times 10^{-7}$, $\beta_{\text{PMMA; 20 keV}} = 3.355 \times 10^{-10}$, $\delta_{\text{PMMA; 25 keV}} = 4.263 \times 10^{-7}$, and $\beta_{\text{PMMA; 25 keV}} = 1.811 \times 10^{-10}$ [5]. The dark-field signal is given by the Fokker–Planck diffusion coefficient as (see Eq. 3 in Ref. [1]): $$D = \frac{p_{\text{Sam.}}^2}{4\pi^2\Delta^2} \log \frac{V_{\text{ref}}}{V_{\text{obs}} + \varepsilon}, \quad (1)$$ where the addition of a small positive term ε regularizes the division. The characteristic length scale in both the carbon-sphere and four-rod images was taken as $p_{\text{Sam.}} = 10$ pixels, and the size of the local-dark-field retrieval window was 10×10 pixels. For the carbon-sphere sample, we found an optimal regularization term of $\varepsilon = 0.55\%$, and for the four-rod sample, $\varepsilon = 1.6\%$.
Single-grid [2]	The grid period was $p_{\text{Grid}} = 8$ pixels, and hence, the correlation window size was $S_{\text{Window}} = 8$ pixels, and moved by $S_{\text{Slide-by}} = 4$ pixels. Analysis was performed at every pixel, meaning $S_{\text{Sample-by}} = 1$ pixel. A median filter with a standard deviation equal to 8 pixels was applied after dark-field retrieval.
Single-speckle [3]	The γ value was taken as that for PMMA for both samples, similar to the dual-energy propagation-based approach above, meaning $\gamma = 2335$ for all reconstructions. The regularization parameter for the inverse transverse Laplacian operator was $0.000\,15\,\mu\text{m}^{-2}$ for both samples, as determined using the approach described in Alloo and Morgan [6] for the multi-speckle approach described below.
Multi-speckle [4]	As above, the *a priori* sample information was taken as $\gamma = 2335$. The Tikhonov regularization parameter in the QR decomposition step was taken as the standard deviation of the coefficient matrix divided by 10^4 (as suggested in Ref [4]). The optimal cut-off parameter required to retrieve an accurate dark-field signal was found to be $\rho = 10\,\mu\text{m}^2$. The Tikhonov regularization parameter for the inverse transverse Laplacian operator was determined to be $0.000\,15\,\mu\text{m}^{-2}$ for both samples, using the iterative approach in Alloo and Morgan [6].

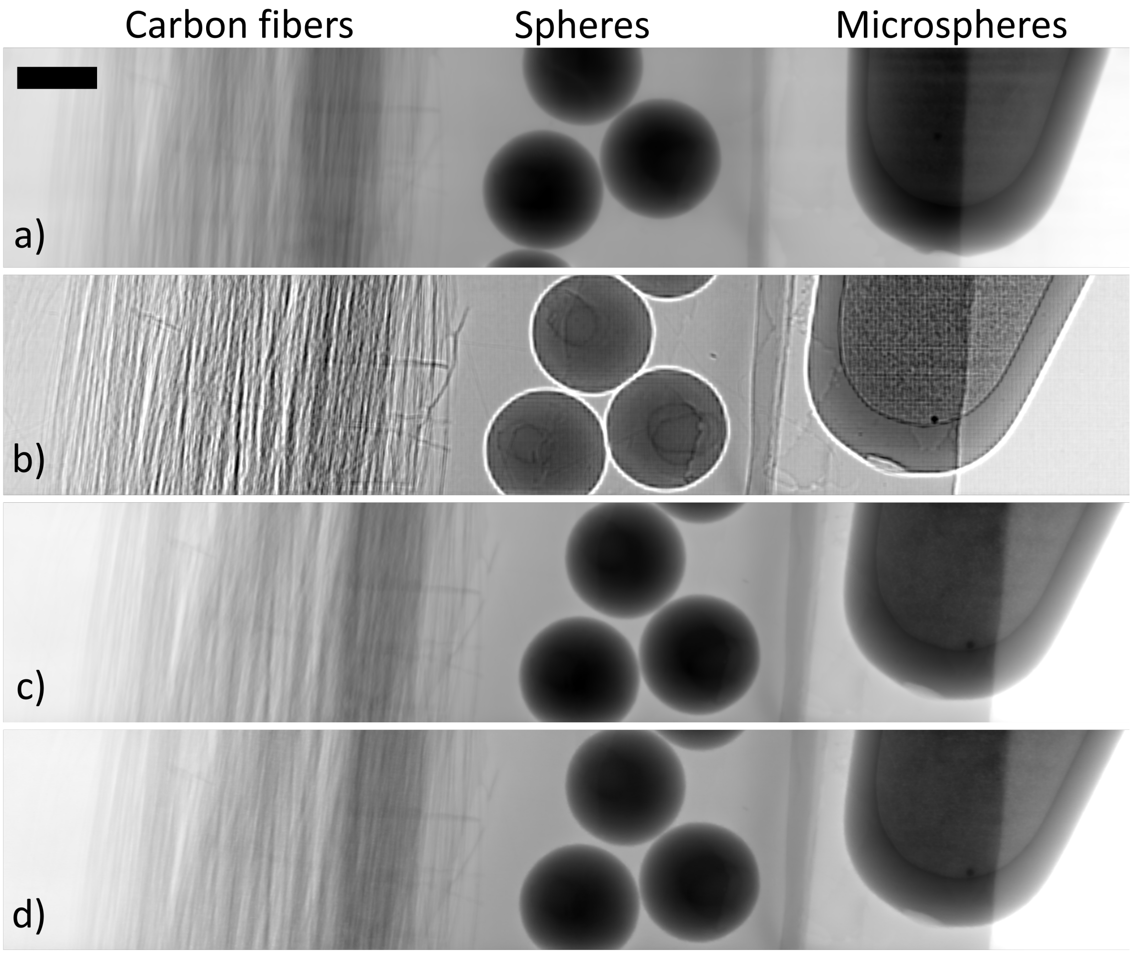

Figure 1: Retrieved transmission images of the carbon-sphere sample using the different retrieval approaches: a) dual-energy propagation-based, b) single-grid, c) single-speckle, and d) multi-speckle. The black rectangle in subfigure a) denotes the 1 mm scale bar for all images. The grayscale ranges for the images are [min (black), max (white)]: a) = [0.86, 0.99], b) = [0.86, 1.00], c) = [0.90, 0.98], and d) = [0.90, 0.98].

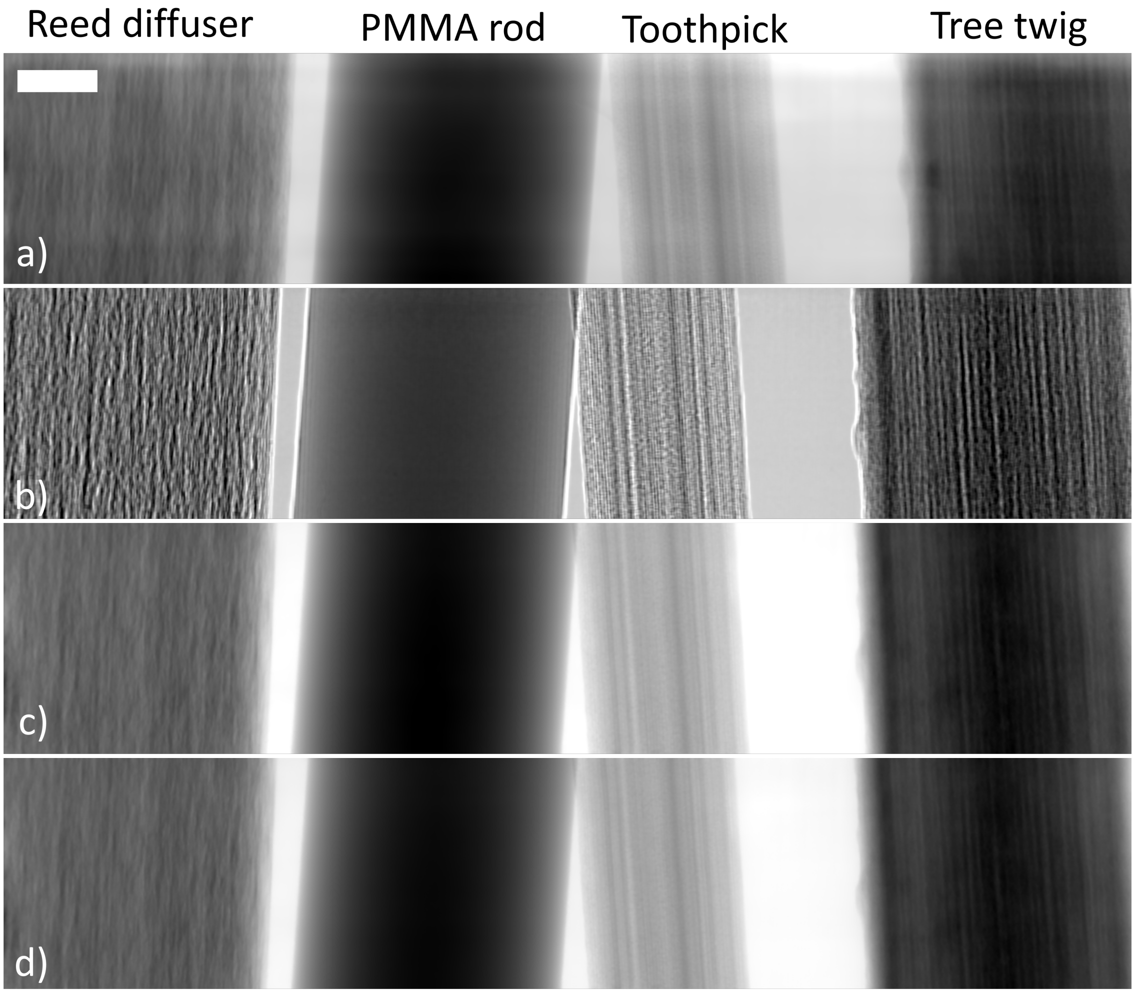

Figure 2: Retrieved transmission images of the four-rod sample using the different retrieval approaches: a) dual-energy propagation-based, b) single-grid, c) single-speckle, and d) multi-speckle. The white rectangle in subfigure a) denotes the 1 mm scale bar for all images.. The grayscale ranges for the images are [min (black), max (white)]: a) = [0.79, 1.00], b) = [0.83, 1.00], c) = [0.87, 1.00], and d) = [0.87, 1.00].

References

[1] J. N. Ahlers, K. M. Pavlov, M. J. Kitchen, and K. S. Morgan, 'X-ray dark-field via spectral propagation-based imaging,' *Optica*, vol. 11, no. 8, pp. 1182–1191, 2024.

[2] Y. Y. How and K. S. Morgan, 'Quantifying the X-ray dark-field signal in single-grid imaging,' *Optics Express*, vol. 30, no. 7, pp. 10899–10918, 2022.

[3] M. A. Beltran, D. M. Paganin, M. K. Croughan, and K. S. Morgan, 'Fast implicit diffusive dark-field retrieval for single-exposure, single-mask X-ray imaging,' *Optica*, vol. 10, no. 4, pp. 422–429, 2023.

[4] S. J. Alloo, K. S. Morgan, D. M. Paganin, and K. M. Pavlov, 'Multimodal intrinsic speckle-tracking (MIST) to extract images of rapidly-varying diffuse X-ray dark-field,' *Scientific Reports*, vol. 13, no. 1, pp. 5424, 2023.

[5] J. H. Hubbell and S. M. Seltzer, *Tables of X-ray mass attenuation coefficients and mass energy-absorption coefficients 1 keV to 20 MeV for elements Z=1 to 92 and 48 additional substances of dosimetric interest*, NISTIR 5632, PB-95-220539/XAB, National Institute of Standards and Technology, Ionizing Radiation Division, Gaithersburg, MD, 1995.

[6] S. J. Alloo and K. S. Morgan, "Stabilizing Laplacian Inversion in Fokker–Planck Image Retrieval using the Transport-of-Intensity Equation," *Physica Scripta*, vol. 100, no. 7, pp. 075566, 2025.
